# 

**Published:** 1875-06

**Authors:** 


### CONTENTS:

### ORIGINAL COMMUNICATIONS.

ARTICLE I.—Notes on Celluloid. By James B. Hodgkin, D. D S.......... 49

ARTICLE II.—Prosecution for Dental Fees.......... 52

ARTICLE III.—Obituary.—Dr. M. McCarty. By S. J. Cobb, D. D. S.......... 62

ARTICLE IV.—What may our Patients Reasonably Expect shall be Accomplished by Operations upon the Teeth. By Geo. H. Cushing, D. D. S.......... 63

ARTICLE V.—A Dentist in the Newspapers.......... 68

ARTICLE VI.—A Biographical Sketch of the Life of Dr. T. B. Hamlin. By S. J. Cobb, D. D. S.......... 72

### SELECTED ARTICLES.

ARTICLE VIII.—An Improved Method of Taking Plaster Impressions. By J. Smith Turner, M. R. C. S.......... 76
American Dental Convention.......... 79
Mississippi State Dental Association.......... 79
The Seventh District Dental Society of N. Y.......... 80
California State Dental Association.......... 80
ARTICLE VIII.—Salicylic Acid. By W. Taft, D. D. S.......... 82
ARTICLE IX.—Monument in Memory of Dr. Horace Wells.......... 84
ARTICLE X.—On the Physiological Action of Arsenious Acid. By Prof. Boehm.... 87

### EDITORIAL, ETC.

Dental Education.......... 89
Dr. Black's Article.......... 93

### MONTHLY SUMMARY.

Treatment of Cold.......... 93

Nelaton's Method of Resuscitation.......... 94

Antidote to Chloroform.......... 94

Solvents for Rubber.......... 95

Singular Death from inhaling Ether.......... 95

Death after Intra-venous Injection of Chloral.......... 96

Intra-venous Injection of Chloral for Producing Anæsthesia.......... 96

